# Pharmacological targeting of valosin containing protein (VCP) induces DNA damage and selectively kills canine lymphoma cells

**DOI:** 10.1186/s12885-015-1489-1

**Published:** 2015-06-24

**Authors:** Marie-Ève Nadeau, Charlène Rico, Mayra Tsoi, Mélanie Vivancos, Sabin Filimon, Marilène Paquet, Derek Boerboom

**Affiliations:** 1 Département des Sciences Cliniques, Université de Montréal, Saint-Hyacinthe, QC J2S7C6 Canada; 2 Département de Biomédecine Vétérinaire, Université de Montréal, Saint-Hyacinthe, QC J2S7C6 Canada; 3 Département de Pathologie et de Microbiologie, Faculté de Médecine Vétérinaire, Université de Montréal, Saint-Hyacinthe, QC J2S7C6 Canada

**Keywords:** Lymphoma, Valosin containing protein, Therapeutic targeting, DNA damage, Apoptosis, Dog

## Abstract

**Background:**

Valosin containing protein (VCP) is a critical mediator of protein homeostasis and may represent a valuable therapeutic target for several forms of cancer. Overexpression of VCP occurs in many cancers, and often in a manner correlating with malignancy and poor outcome. Here, we analyzed VCP expression in canine lymphoma and assessed its potential as a therapeutic target for this disease.

**Methods:**

VCP expression in canine lymphomas was evaluated by immunoblotting and immunohistochemistry. The canine lymphoma cell lines CLBL-1, 17–71 and CL-1 were treated with the VCP inhibitor Eeyarestatin 1 (EER-1) at varying concentrations and times and were assessed for viability by trypan blue exclusion, apoptosis by TUNEL and caspase activity assays, and proliferation by propidium iodide incorporation and FACS. The mechanism of EER-1 action was determined by immunoblotting and immunofluorescence analyses of Lys48 ubiquitin and markers of ER stress (DDIT3), autophagy (SQSTM1, MAP1LC3A) and DNA damage (γH2AFX). TRP53/ATM-dependent signaling pathway activity was assessed by immunoblotting for TRP53 and phospho-TRP53 and real-time RT-PCR measurement of *Cdkn1a* mRNA.

**Results:**

VCP expression levels in canine B cell lymphomas were found to increase with grade. EER-1 treatment killed canine lymphoma cells preferentially over control peripheral blood mononuclear cells. EER-1 treatment of CLBL-1 cells was found to both induce apoptosis and cell cycle arrest in G1. Unexpectedly, EER-1 did not appear to act either by inducing ER stress or inhibiting the aggresome-autophagy pathway. Rather, a rapid and dramatic increase in γH2AFX expression was noted, indicating that EER-1 may act by promoting DNA damage accumulation. Increased TRP53 phosphorylation and *Cdkn1a* mRNA levels indicated an activation of the TRP53/ATM DNA damage response pathway in response to EER-1, likely contributing to the induction of apoptosis and cell cycle arrest.

**Conclusions:**

These results correlate VCP expression with malignancy in canine B cell lymphoma. The selective activity of EER-1 against lymphoma cells suggests that VCP will represent a clinically useful therapeutic target for the treatment of lymphoma. We further suggest a mechanism of EER-1 action centered on the DNA repair response that may be of central importance for the design and characterization of VCP inhibitory compounds for therapeutic use.

**Electronic supplementary material:**

The online version of this article (doi:10.1186/s12885-015-1489-1) contains supplementary material, which is available to authorized users.

## Background

Canine lymphoma shares many similarities with human non-Hodgkin’s lymphoma (NHL) with respect to its molecular and clinical features [1, 2]. It is one of the most common neoplasms in dogs and its incidence is reported to be on the rise, at more than 33 diagnoses per 100 000 dog-years in 2002 [3]. Dogs will usually present with rapidly progressing, high grade, multicentric disease in an advanced stage (III or IV/V). Not unlike humans, the most common histologic subtype diagnosed is diffuse large B cell lymphoma [4]. First line treatment is a “CHOP”-based (cyclophosphamide, doxorubicin, vincristine, corticosteroids) chemotherapy protocol, which results in a complete response of 6 to 11 months duration in greater than 80 % of cases. However, overall survival for dogs with lymphoma remains brief, and averages 12 months with approximately 10 % surviving 2 years [5]. As is the case for human NHL, chemoresistance occurring either at onset or at recurrence is a main reason why treatment ultimately fails [6, 7]. The many similarities as well as the rapid course of disease make canine lymphoma an attractive model for the study of novel therapeutics for NHL.

Several strategies are currently being investigated to circumvent chemoresistance. One that seems to hold particular promise is the development of molecular-targeted therapies based on the molecular pathways that drive NHL cell proliferation and survival [1, 5, 6]. Among the druggable targets currently under investigation in the pharmaceutical industry is valosin containing protein (VCP, also known as p97). VCP is a member of the AAA family of ATPases and is a critical mediator of protein homeostasis [8]. Through its interaction with several accessory proteins and cofactors, VCP notably mediates endoplasmic reticulum-associated degradation (ERAD), the process by which misfolded proteins localized in the ER lumen or membrane are eliminated. Following their ubiquitination, VCP is thought to extract the targeted proteins from the ER in an ATP-dependent manner, and maintain their misfolded state until they can be degraded by the cellular proteasomal machinery [9]. VCP is also involved in the aggresome-autophagy pathway, which is required for the clearance of misfolded proteins that form aggregates in the cytosol. Here, VCP may act to recruit E4B ubiquitin ligase activity to aggregates of misfolded proteins [10] and/or mediate the fusion of misfolded protein-containing autophagosomes with lysosomes [11]. More recently, VCP has also been associated with the degradation of chromatin-associated proteins, including those involved in processes such as DNA replication and repair, cell division, and gene transcription [12, 13]. Inhibition of VCP activity therefore has a range of consequences for the cell, beginning with the accumulation of misfolded, polyubiquitinated proteins and culminating in apoptosis, often triggered by ER stress and the unfolded protein response [14, 15]. Due to their higher metabolic and proliferative rates, cancer cells require increased activities of ER machinery in facilitating protein folding, assembly, and transport, and are therefore thought to be more reliant on VCP for the clearance of misfolded proteins that their normal counterparts [16]. This is supported by the documented overexpression of VCP in many cancers including lymphoma, and often in a manner correlating with malignancy and poor outcome [17–20].

Evidence is accumulating that suggests that VCP represents a valid therapeutic target for a range of cancers. Of particular relevance to the present study, the VCP inhibitor Eeyarestatin 1 (EER-1) was shown to have a strongly preferential cytotoxic activity against various human haematological cancer cell lines, relative to peripheral blood mononuclear cells (PBMCs) [15]. EER-1 also inhibited tumor growth in a mouse non-small cell lung cancer xenograft model, without overt side effects [21]. These studies, coupled with the ongoing development of small molecule inhibitors of VCP intended for therapeutic use [22, 23], indicate that VCP will represent a major target in the development of the next generation of cancer treatments.

The aim of the present study was to evaluate VCP as a therapeutic target for lymphoma using the canine model. Here, we show that VCP expression correlates with malignancy in canine B-cell lymphoma. We further demonstrate that pharmacological inhibition of VCP results in preferential lymphoma cell kill over PBMCs, validating VCP as a therapeutic target. Unexpectedly, we also found evidence suggesting that EER-1 induces apoptosis in CLBL-1 cells via the accumulation of DNA damage rather than by the induction of ER stress. These findings will serve as the conceptual basis for the design of clinical trials using VCP inhibitory compounds for the treatment of lymphoma.

## Methods

### Tumor samples

Frozen and formalin-fixed lymphoma tumor samples were obtained from the Canine Comparative Oncology and Genomics Consortium and from the Oncology Service at the Faculté de Médecine Vétérinaire, Université de Montréal. All tumor grades were determined by a single pathologist (MP) using the classification system established by Valli et al. [4]. Immunophenotype was determined through CD3 and CD79a immunohistochemistry. Lymph nodes used as controls were from cadavers of healthy dogs euthanized for reasons unrelated to illness, and were obtained from the Département de Pathologie et de Microbiologie, Faculté de Médecine Vétérinaire, Université de Montréal.

### Immunohistochemistry

Immunohistochemistry was done on formalin-fixed, paraffin-embedded, 3 μm lymphoma and normal lymph node sections using the VectaStain Elite Avidin-Biotin Complex Kit (Vector Laboratories, Inc.) as directed by the manufacturer. Sections were probed with anti-VCP mouse monoclonal antibody (Abcam Inc., catalog number ab11433, dilution 1:4000) as directed by the manufacturer, except blocking was done with 5 % normal serum in TBST for 1 h at room temperature and incubation with the secondary antibody (biotinylated anti-mouse reagent, Vector Laboratories, Inc., dilution 1:500) was done for 30 min. Staining was done using 3,3’-diaminobenzidine peroxidase substrate kit (Vector Laboratories, Inc.) as directed by the manufacturer. Negative controls were done using the primary antibody described above that was pre-incubated overnight at 4 °C with VCP peptide (792–806, Abcam Inc., catalog number ab39788) in a 1:10 antibody:peptide ratio.

### Cell culture

The cell lines used in this study (CL-1, 17–71, CLBL-1) have been previously characterized and were cultured as previously described [24, 25]. Briefly, cells were grown in T75 flasks using RPMI medium (Invitrogen) containing 10 % (CL-1 and 17–71) or 20 % (CLBL-1) heat inactivated fetal bovine serum (FBS, Invitrogen), 100 units/ml of penicillin, 100 μg/ml of streptomycin and 0.25 μg/ml of fungizone (Invitrogen), and incubated at 37 °C in humidified 5 % CO_2_/95 % air.

Peripheral blood mononuclear cells (PBMCs) were isolated from normal dogs using Histopaque-1077 (10,771; Sigma) according to the manufacturer’s recommendations. Briefly, whole blood was collected in heparinized tubes, layered on an equal volume of histopaque-1077 and centrifuged at 400 g for 30 min for the recovery of mononuclear cells. PBMCs were cultured under the same conditions as CL-1 and 17–71 cells (as described above). All animal procedures were approved by the Institutional Animal Care and Use Committee of the Université de Montréal and were in accordance with the Canadian Council on Animal Care (CCAC) policy on humane care and use of laboratory animals.

### Dose response experiment

Cells were seeded in 24-well plates at a density of 50 × 10^3^ cells per well for 17–71, CL-1 and CLBL-1 cells or 250 × 10^3^/well for PBMCs, and treated with vehicle (DMSO) or graded doses of Eeyarestatin 1 (EER-1, #324,521; Calbiochem) for 48 h (n = 3 wells/treatment). The number of viable cells was counted 3 times per well using the trypan blue exclusion assay and a hemocytometer [26]. The number of viable cells in the treated groups was then normalized to the number of viable cells in the control group (vehicle). This experiment was repeated 3 times.

### Time course analyses

CLBL-1 cells were seeded at a density of 2 × 10^6^ cells per well in a 6-well tissue culture plate and treated with vehicle (DMSO) or 3 μM EER-1 for 6, 12, or 24 h (n = 3 per time point). Cells were then either (i) collected for protein or mRNA extraction, (ii) fixed for immunofluorescence analysis, or (iii) used for flow cytometry or apoptosis analyses (see below). All experiments were repeated 3 times.

### Apoptosis assays

TUNEL assay: Apoptosis was detected using the In Situ Cell Death Detection Kit, TMR red (#12,156,792,910; Roche), following manufacturer’s instructions for cells grown in suspension. Apoptotic cells were imaged using an Axio Imager M.1 microscope (Zeiss) and AxioVision 4.6.3 software. For each sample, 3 photomicrographs of random fields were taken at 200× magnification, and cells were scored as apoptotic or viable and counted.

Caspase 3/7 assay: The Caspase-Glow (#G8090; Promega) assay kit was used following manufacturer’s instructions. Briefly, for each sample 75 μl of Caspase-Glo 3/7 reagent was added to 75 μl of cultured cells (≈20 × 10^3^ cells) in a 96-well plate. The plate was incubated at room temperature for 3 h prior to quantification using a plate-reading luminometer (SpectraMax i3, Molecular Devices).

### Cell cycle analysis

CLBL-1 cells were washed twice with PBS, counted and resuspended at a concentration of 10^6^ cells/ml in Krishan buffer: 0.1 % Sodium Citrate, 0.02 mg/ml Rnase (DNase free), 0.3 % NP-40 and 0.05 mg/ml propidium iodide. Cells were incubated at least 30 mininutes on ice in the dark before being analyzed on an Accuri C6 flow cytometer (BD Biosciences), using BD Accuri C6 software version 1.0.264.21. Cells were gated according to a 2-parameter dot-plot: FL2-A (area) vs Width to monitor doublets. Cell cycle analysis was performed using a single-parameter histogram (FL2-A) with linear x-axis to represent DNA content.

### Immunoblot analysis

Proteins were extracted using M-PER® mammalian protein extraction reagent (#78,501; Thermo Scientific) according to the manufacturer’s instructions. Proteins were quantified using the Bradford method (BIO-RAD Protein Assay, 500–0006). Samples (15 μg) were resolved on 12 % sodium dodecyl sulfate-polyacrylamide gels and transferred to Hybond-P PVDF membrane (GE Amersham). Blots were then probed at 4 °C overnight with antibodies against γH2AFX (#ab26350; Abcam, 1/1000), Lys48 Ubiquitin (#05-1307, Millipore, 1/2000), SQSTM1 (#ab56416; Abcam, 1/1000), DDIT3 (#ab11419; Abcam, 1/100), MAP1LC3A (#4599, Cell signaling, 1/1000), phospho-TRP53 (#9284, Cell signaling, 1/1000), TRP53 (#ab26; Abcam, 1/1000) or ACTB (#sc-8432; Santa Cruz, 1/50000). ACTB was used as the loading control. Following incubation with horseradish peroxidase-conjugated secondary anti-rabbit or anti-mouse antibody, the protein bands were visualized by chemiluminescence using the Immobilon Western HRP substrate (#WBKLS0500, Millipore). Signals were visualized on a Bio-Rad ChemiDoc MP imaging system and quantified using Image Lab 5.0 software (Bio-Rad laboratories).

Proteins for immunoblot analyses of VCP expression in lymphoma and healthy nodes were extracted using RIPA buffer, PhosSTOP Phosphatase Inhibitor Cocktail Tablet and Complete Mini Protease Inhibitor Cocktail Tablet (Roche Diagnostics, Indianapolis, IN, catalog numbers 04906845001 and 11836153001, respectively). Blots were prepared as described above using 18 μg of protein for each sample, and probed with a primary antibody against VCP (Abcam Inc., number ab11433). Subsequent detection and quantification steps were as described above.

### Immunofluorescence

Cells were washed once with PBS and fixed in 2 % paraformaldehyde for 1 h at room temperature. Permeabilization was done with 0.1 % Sodium Citrate, 0.1 % Triton X-100 for 2 mininutes on ice. Cells were incubated for 1 h with a blocking solution (PBS with 10 % goat serum) at room temperature prior to sequential addition of γH2AFX (1/500) and Lys48 Ubiquitin (1/500) antibodies for 1 h at room temperature. Secondary anti-mouse (Alexa fluor 488, Invitrogen) and anti-rabbit (Alexa fluor 594, Invitrogen) antibodies were added simultaneously (1/500) for 1 h at room temperature in the dark. Slides were mounted using Vectashield with 4’,6-diamidino-2-phenylindole (DAPI, Vector Laboratories). Negative controls were run omitting the primary antibody. Images were taken using an Axio Imager M.1 microscope (Zeiss) and analyzed using Zen software.

### Real-time PCR

Total RNA was extracted using the Rneasy mini Kit (#74106, Qiagen) and 200 ng of total RNA were reverse-transcribed using the SuperScript Vilo cDNA Synthesis kit (#11754, Invitrogen) following the manufacturer’s instructions. Real-time PCR reactions were run on a C1000 Touch thermal cycler (Bio-Rad laboratories) using SsoAdvanced Universal SYBR Green Supermix (#172-5274, Bio-Rad laboratories). Each PCR reaction consisted of 7.5 μl of SYBR Green Supermix, 2.3 μl of water, 0.6 μl of gene-specific primers (10pmol) and 4 μl of diluted cDNA sample (1/10). PCR reactions run without cDNA (water blank) served as negative controls. A common thermal cycling program (3 mininutes at 95 °C, 40 cycles of 15 secondes at 95 °C, 30 secondes at 60 °C and 30 secondes at 72 °C) was used to amplify each transcript. A melting curve analysis and gel electrophoresis were also done to ensure that a single PCR product was amplified with each primer pair. Efficiency curves were generated using serial dilutions of cDNA in abscissa and the corresponding cycle threshold in ordinate. The slope of the log-linear phase reflects the amplification efficiency (E) derived from the formula E = e^(1/slope)^. To quantify relative gene expression, the Ct of target gene amplification was compared to that of the internal reference gene *RPL19*, according to the ratio R = [E^CtL19^/E_target_^Ct target^]. Verification tests were done in accordance with MIQE guidelines. Primer sequences were: *Rpl19* sense 3’- TCCAGTGTCCTCCGCTGTGGCAAA-5’; antisense 3’-TTCCGGCGGGCCAGAGTGTTTTT-5’; *Cdkn1a* sense 3’-GATTCGCGGAGCCGGAG-5’; antisense 3’- TTGCTGCCATGAGGGATGG-5’.

### Statistical analyses

The cell viability and TUNEL experiments were analyzed using two-way ANOVA with the Newman-Keuls post-test. All other data were analyzed using unpaired t-tests. Data was log-transformed whenever variances were significantly different between samples. Differences were considered significant when *P* < 0.05.

## Results

### VCP expression correlates with malignancy in canine B cell lymphomas

**Fig. 1:**
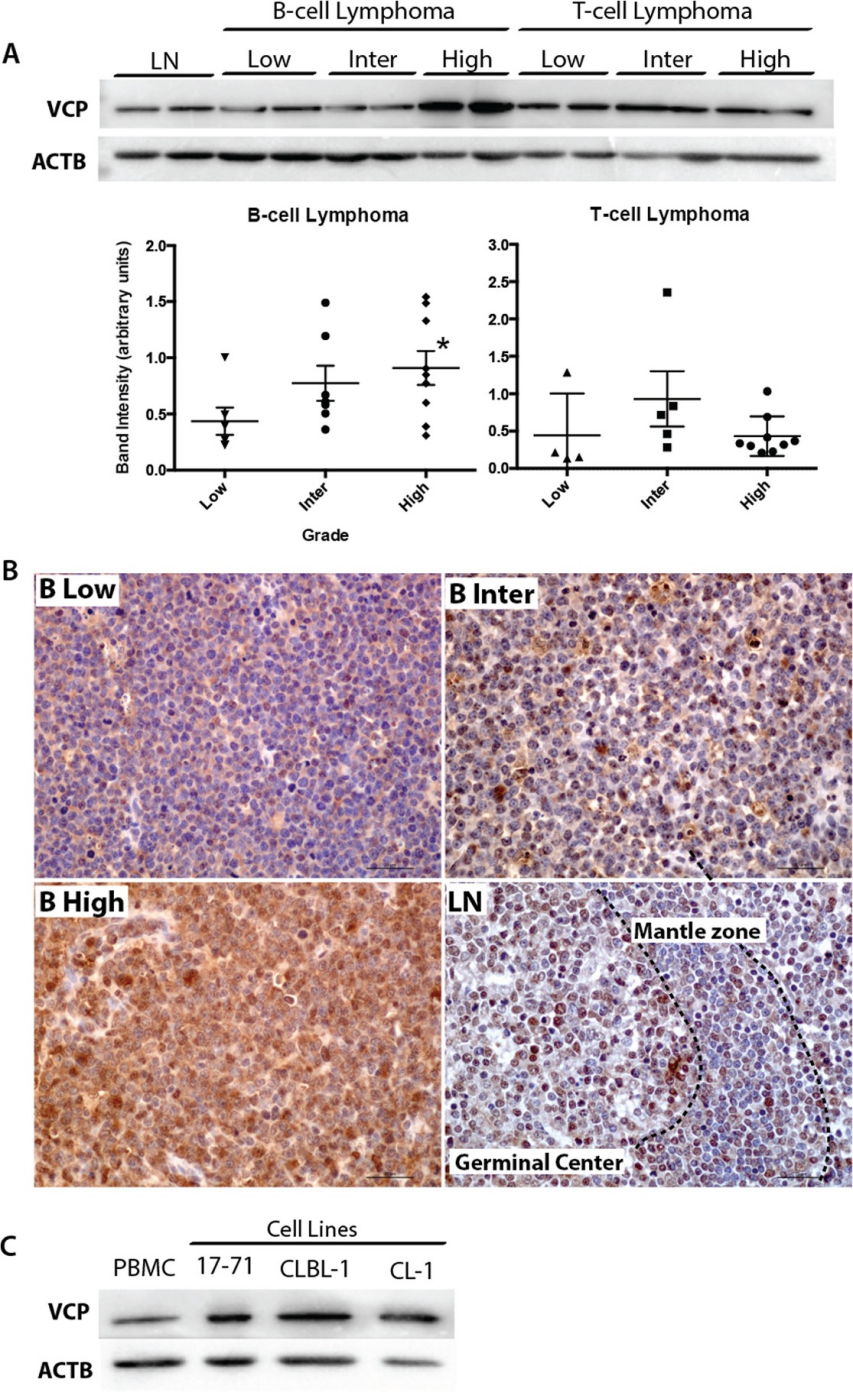
VCP protein expression in canine lymphoma. **a** Immunoblotting analysis was done for VCP. Representative blots are shown, each lane represents a single lymph node or tumor sample from one patient. Quantitative analyses of were done using n = 4–9 tumor samples per grade and type and normalized to ACTB (β actin) as a loading control. Data are presented as mean (cross bar) ± SEM (error bars). Asterisk (*) indicates a statistically significant difference (*P < 0.05) compared to low grade. **b** VCP immunohistochemistry for B-cell lymphoma compared to normal lymph node. **c** Immunoblotting analysis of VCP in PBMCs (signal intensity = 1.01 arbitrary units) and lymphoma cell lines (17–71, CL-1 and CLBL-1) (average signal intensity = 3.33 arbitrary units). High, Inter (Intermediate) and Low refer to grade, T and B refer to lymphoma cellular subtype, LN = normal lymph node

To study VCP expression in canine lymphoma, tumor VCP protein levels were analyzed by immunoblotting and compared to normal lymph nodes. Whereas low grade B-cell lymphomas were found to express VCP at levels comparable to normal lymph nodes, significantly higher VCP expression was found in high grade B-cell lymphomas (Fig. 1a). Immunohistochemical analyses confirmed these findings and further showed that low grade B-cell lymphomas express VCP at a level comparable to lymphocytes present in the mantle zone of lymphoid follicles, whereas VCP expression in medium and high grade B-cell lymphomas was more comparable to that found in lymphocytes of germinal centers (Fig. 1b). VCP expression in T-cell lymphomas on the other hand did not vary significantly according to grade (Fig. 1a). VCP expression levels in canine lymphoma cell lines were also analyzed by immunoblotting and compared to PBMCs. Analyses included the B-cell lymphoma-derived lines CLBL-1 and 17–71, and the T-cell lymphoma line CL-1. VCP expression was found to be to be higher in all lymphomas cell lines compared to PBMCs (Fig. 1c).

### Lymphoma cells have an increased sensitivity to VCP inhibition

**Fig. 2:**
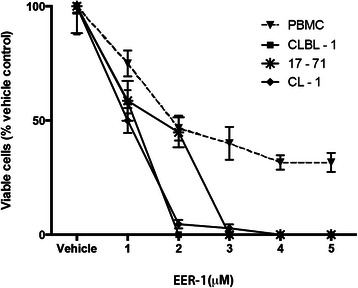
VCP inhibition decreases canine lymphoma cell viability. PBMCs, CLBL-1, 17–71 and CL-1 cells were treated for 48 h with graded doses of EER-1. Cell viability was evaluated by trypan blue exclusion. At 1 and 2 μM doses, the proportion of viable PBMCs was significantly higher than in CL-1 cells (*p* < 0.05 and *p* < 0.01, respectively). At 3, 4 and 5 μM, the proportion of viable PBMCs was significantly higher than CLBL-1, 17–71 or CL-1 cells (*p* < 0.001 for each cell line and for each inhibitor concentration). Data are presented as means ± SEM (error bars), *n* = 4 replicates per treatment. The experiment was repeated three times, and a representative result is shown

To determine if VCP inhibition would affect lymphoma cells differently than their normal counterparts, CLBL-1, CL-1, 17–71 cell lines as well as PBMCs were cultured for 48 h and exposed to increasing concentrations of EER-1. Whereas even the highest tested dose failed to reduce numbers of viable PBMCs below ~40 % of control, virtually all lymphoma cells were killed by 2 μM (CLBL-1 and CL-1) or 3 μM (17–71) EER-1 (Fig. 2).

**Fig. 3:**
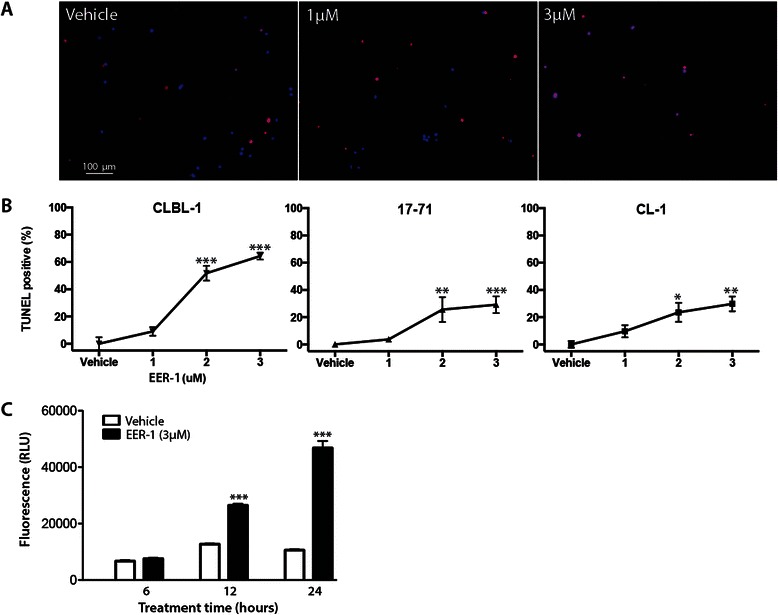
VCP inhibition induces apotosis in canine lymphoma cell lines. **a** Representative images of TUNEL assays done on CLBL-1 cells following treatment with vehicle (left panel), 1 μM (middle panel) and 3 μM (right panel) of EER-1 treatment. Live cells appear blue (DAPI signal) and an apoptotic cells appear purple (merged red TUNEL signal + DAPI). **b** Quantitative analysis of the TUNEL assays . **c** Caspase 3/7 activity assay. Data are presented as means (columns) ± SEM (error bars). Asterisks indicate a statistically significant difference (*P < 0.05; **P < 0.01 or ***P < 0.001) compared to the vehicle. For both experiments, *n* = 3 replicates per treatment, each experiment was repeated three times and a representative result is shown

To determine if EER-1 treatment induced apoptosis in lymphoma cells, TUNEL assays were done on cultured cells treated with 1, 2 or 3 μM EER-1 for 48 h. Significant increases in the number of TUNEL-positive cells was noted in all cell lines at doses of 2 μM or greater, with CLBL-1 cells being the most sensitive to EER-1 treatment (Fig. 3a, b). To further analyze the kinetics of induction of apoptosis by EER-1, CLBL-1 cells were cultured for 6, 12 and 24 h with or without 3 μM EER-1, and the activation of the caspase cascade determined using the Caspase-Glo 3/7 assay. By this method, EER-1 was found to induce apoptosis as early as 12 h post-treatment (Fig. 3c).

**Fig. 4:**
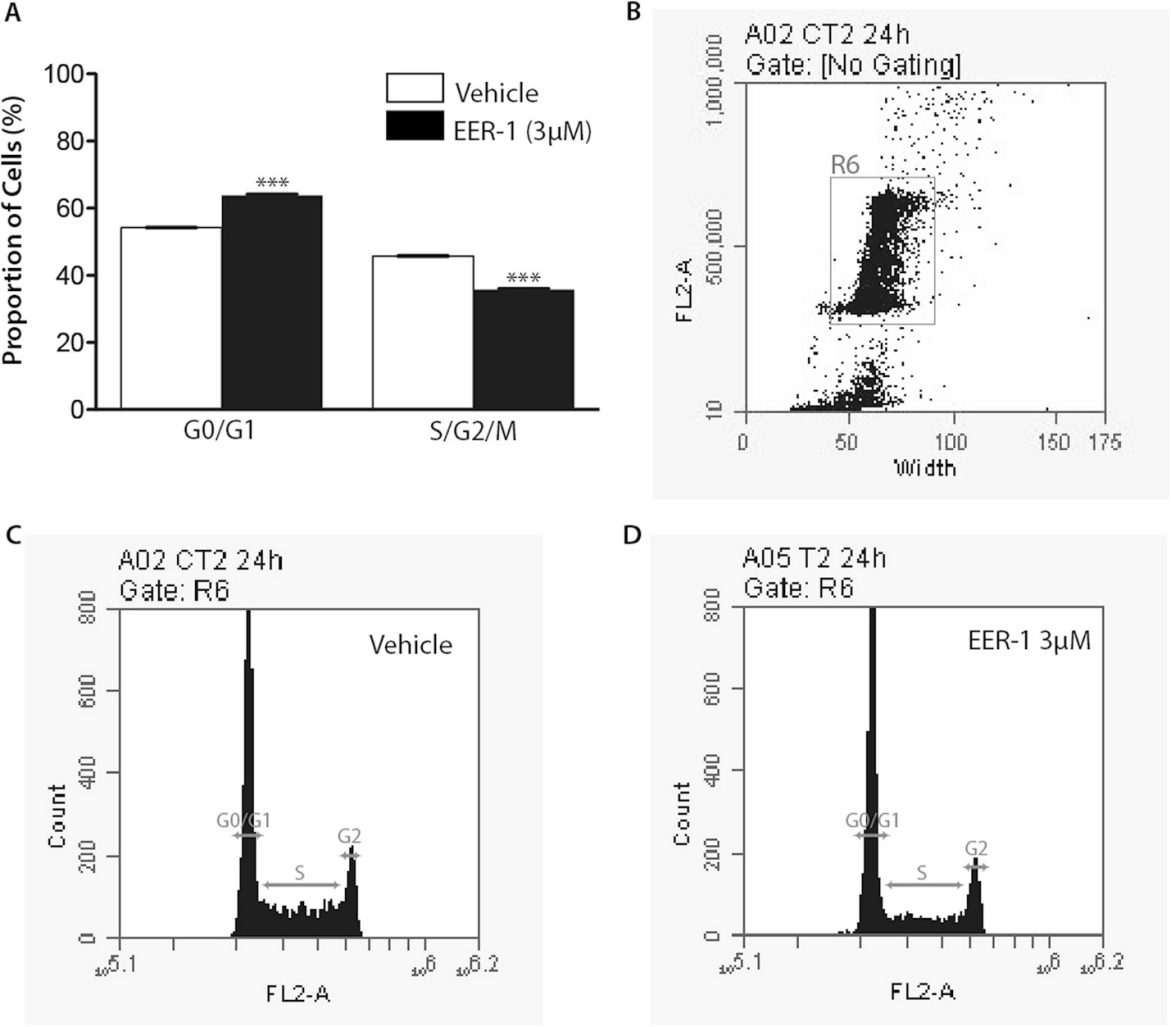
VCP inhibition results in G1 cell cycle arrest*.***a** Propidium iodide incorporation/FACS analysis of vehicle-treated (white columns) or EER-1-treated (black columns) cells. Cells were gated and counted as described below and in the Methods section and grouped into the G0/G1 or in S/G2/M phases. Data are presented as mean (columns) ± SEM (error bars), *n* = 3 analyses/treatment. Asterisks indicate a statistically significant difference (*P* < 0.001) from vehicle-treated group. The experiment was repeated three times and a representative result is shown. **b** Representative two parameter dot-plot (FL2-A (area) vs width); the gray rectangle represents the population of cells analyzed in the FACS experiment. Representative FACS analyses of vehicle-treated (**c**) and EER-1-treated (**d**) cells. Gray arrows indicate the populations that were defined as G0/G1, S and G2/M

To assess the effects of VCP inhibition on cell proliferation, cell cycle distribution was determined in CLBL-1 cells at different times following EER-1 treatment. Propidium iodide incorporation and FACS analyses showed no effect of EER-1 after 6 or 12 h of treatment (not shown), but after 24 h the proportion of cells in the S and G2/M phases was reduced, as evidenced by decreased heights of the G2/M peak and the S-phase plateau between the G0/G1 and G2/M peaks (Fig. 4, quantification shown in 4a). This was accompanied by a modest (c.5 %) but significant increase in G0/G1 peak (Fig. 4).

### VCP inhibition results in DNA damage and TRP53 pathway activation

**Fig. 5:**
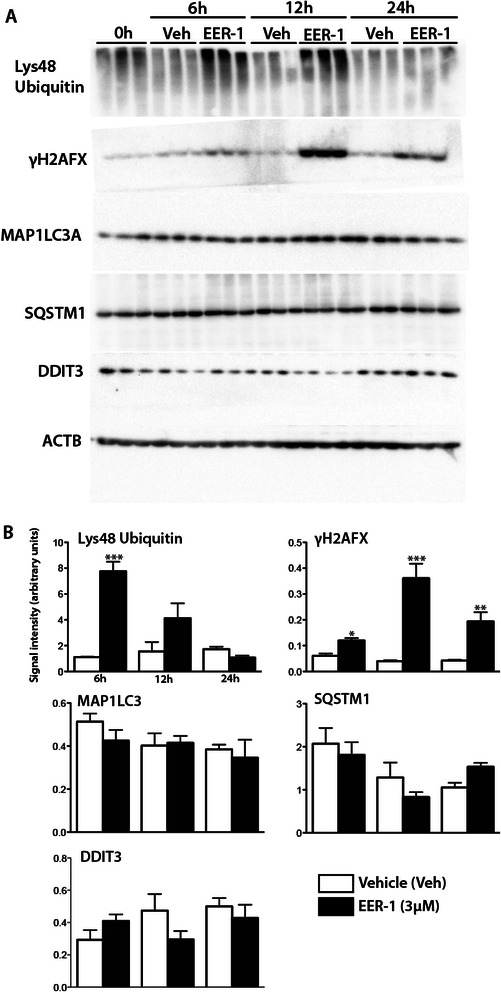
VCP inhibition induces concomitant increases in Lys48 polyubiquitinated proteins and γH2AFX. **a**) Immunoblotting analysis was done for Lys48 ubiquitin, γH2AFX, MAP1LC3A, SQSTM1and DDIT3 on CLBL-1 cells treated on a time course with 3 μM EER-1. Representative blots are shown, each lane represents cells collected from a single well. **b**) Quantitative analyses of were done using *n* = 3 replicates per condition and normalized to ACTB (β actin) as a loading control. Data are presented as means (columns) ± SEM (error bars). Asterisks indicate a statistically significant difference (*P < 0.05, **P < 0.01 and ***P < 0.001) compared to their respective control. Veh = Vehicle. The experiment was repeated three times, and a representative result is shown

**Fig. 6:**
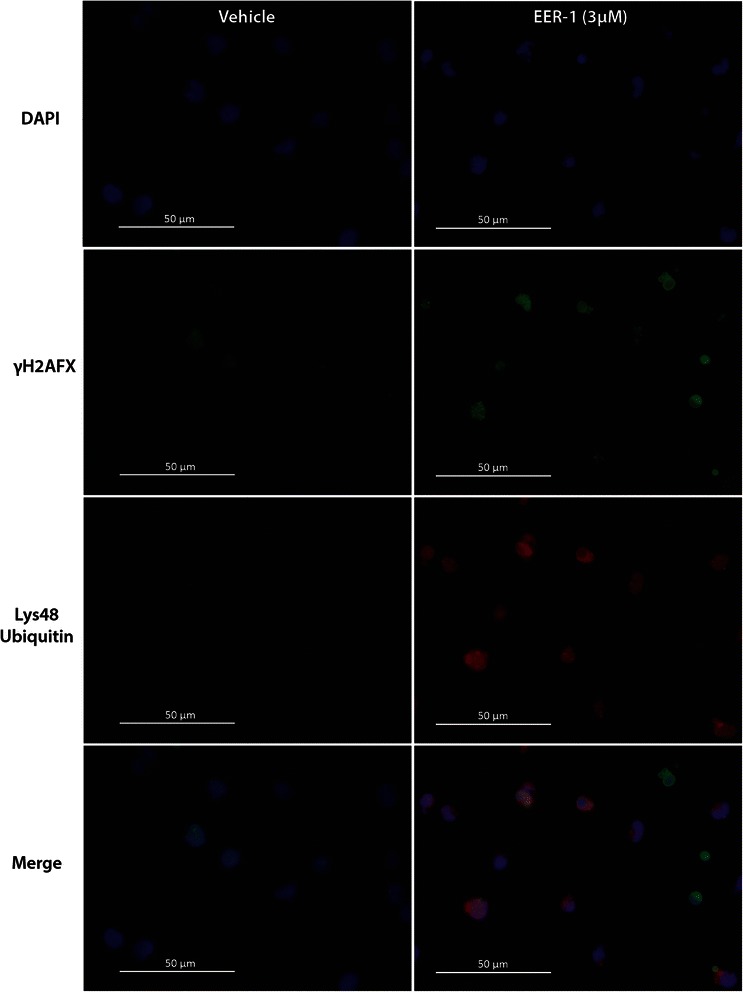
VCP inhibition results in nuclear Lys48 ubiquitin protein accumulation and DNA damage. Cells were cultured for 12 h with or without 3 μM EER-1. Co-immunolabelling was performed for Lys48 ubiquitin and γH2AFX for treated (right panels) and untreated cells (left panels). A negative control was performed without the primary antibody (not shown)

VCP is central to cellular protein homeostasis [8]. As such it is involved in many aspects of cellular protein degradation including endoplasmic reticulum-associated degradation (ERAD), the aggresome-autophagy pathway (AA) and chromatin-associated degradation (CAD). To determine if the cytotoxicity of EER-1 in lymphoma cells could be specifically associated with perturbations in any of the aforementioned processes, CLBL-1 cells were cultured and treated with EER-1 over a time course. Immunoblotting was then done to assess levels of Lys48 polyubiquitinated proteins, as well as markers of ERAD (DDIT3), AA (SQSTM1, MAP1LC3A) and DNA damage (γH2AFX, a marker of double-stranded DNA breaks). As expected, EER-1 treatment resulted in the accumulation of polyubiquitinated proteins with peak levels observed 6 h post-treatment (Fig. 5), reflecting decreased proteosomal degradation. Surprisingly however, no alterations in DDIT3, SQSTM1 or MAP1LC3A were detected, whereas the positive controls thapsigargin and chloroquine readily induced DDIT3 and MAP1LC3A(II) expression, respectively (Additional file 1: Figure S1). These findings suggest that the cytotoxic effects of EER-1 were not the result of ERAD or AA inhibition. Conversely, a dramatic increase in γH2AFX levels was noted at all time points, attaining peak levels at 12 h following EER-1 treatment. Immunofluorescence analyses of CLBL-1 cells confirmed the increase in γH2AFX expression in response to EER-1, and in a manner coincident with the accumulation of Lys48 polyubiquitinated proteins in both the cytoplasm and nucleus (Fig. 6).

**Fig. 7:**
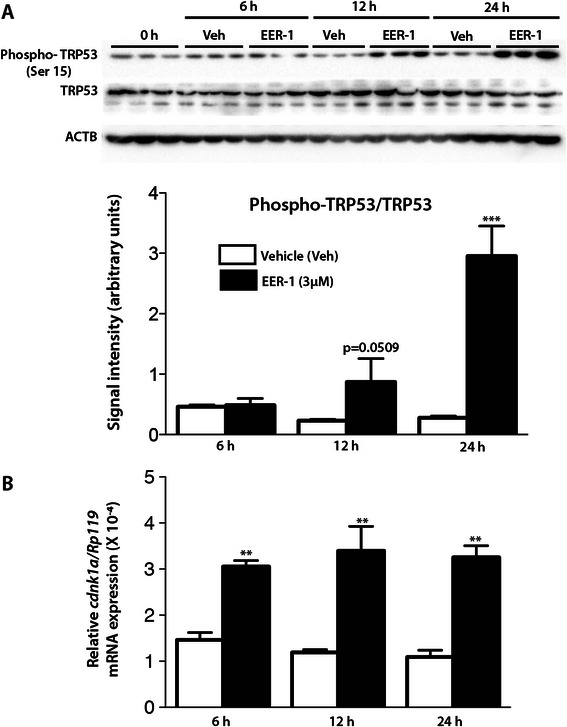
EER-1 treatment results in TRP53 pathway activation in canine lymphoma cells. CLBL-1 cells were cultured for 6, 12 or 24 h with or without 3 μM EER-1. **a** Immunoblotting analysis for phospho-TRP53 (Ser 15) and total TRP53. Representative blots are shown (upper panels), each lane represents cells from a single well. Quantitative analyses of phospho-TRP53/ total TRP53 ratios (lower panel) were done using *n* = 3 replicates per condition. **b***Cdkn1a* mRNA expression was analyzed by real time PCR. Data are presented as mean (columns) ± SEM (error bars). Asterisks indicate a statistically significant difference (**P < 0.01 and ***P < 0.001) compared to their respective control. The experiment was repeated three times, and representative results are shown

As these results suggested that EER-1-induced apoptosis in CLBL-1 cells may be the direct result of DNA damage, we determined if EER-1 treatment activates the TRP53/ATM-dependent signaling pathway. In the latter, the kinase ATM is recruited to double-stranded DNA breaks and phosphorylates a range of substrates including the tumor suppressor protein TRP53. Phospho-TRP53 in turn participates in the transcriptional activation of the cyclin-dependent kinase inhibitor *Cdkn1a*, resulting in cell cycle arrest G1/S checkpoint or apoptosis [27]. CLBL-1 cells were cultured and treated with EER-1 in a time course experiment, and TRP53 (Ser15) phosphorylation was evaluated by immunoblotting and *Cdkn1a* expression was evaluated by qRT-PCR. Both phosphoTRP53 and the ratio of phospho:total TRP53 increased progressively in response to EER-1, attaining statistical significance at 24 h post-treatment (Fig. 7a). *Cdkn1a* mRNA levels were also increased in the treated group compared to control at all time points examined (Fig. 7b).

## Discussion

A number of studies so far have examined VCP expression in human malignancies [18–20, 28–33], but only Zhu et al. have specifically studied lymphoma [17]. In the latter report, VCP expression levels in primary orbital MALT lymphoma (a type of B-cell lymphoma) were found to correlate in a positive manner with disease recurrence and in a negative manner with patient survival [17]. Here, we show for the first time that increased VCP expression also occurs in canine B-cell lymphoma, specifically in high-grade forms of the disease. The biological significance of this finding remains to be determined, but could indicate that malignant B-cell lymphomas produce greater amounts of polyubiquitinated and misfolded proteins than normal B cells, therefore requiring increased levels of VCP expression to ensure their proteosomal degradation, reduce ER stress and avoid undergoing apoptosis. Why malignant B cells would produce more polyubiquitinated and misfolded proteins is also unclear, but could simply be a by-product of their increased secretory, metabolic and proliferative activity. Indeed, others have suggested that the secretory demands that come with B-cell differentiation may result in a basal level of ER stress and unfolded protein response activation [34–36]. Furthermore, we found VCP expression in high-grade B-cell lymphomas to be comparable to that found in the germinal centers of lymph nodes, which represent a highly proliferative subpopulation of B cells [37]. Conversely, VCP expression in low grade B-cell lymphomas was comparable to that found in the (more differentiated and less proliferative) B cells that compose the mantle zone. VCP expression may therefore reflect both the malignancy and the proliferative activity of B-cell lymphomas, and may be predictive of their responsiveness to VCP-targeted therapies. The latter theory appears to be supported by our pharmacological studies, as the B-cell lymphoma lines 17–71 and CLBL-1 had increased VCP expression and were far more sensitive to VCP inhibition than normal blood mononuclear cells.

Given the nature of its biological functions, several authors have proposed that VCP could represent a pharmacological target for the treatment of cancer [8, 12, 16, 21, 38]. Indeed, several novel VCP-inhibitory compounds have recently been reported [14, 22, 23, 39–41] and are currently under development for therapeutic use. The first indication that VCP inhibitors could be useful against lymphoma was a study by Wang et al., which showed that EER-1 has a strongly preferential cytotoxic activity against several human haematological cancer cell lines (including mantle cell and Burkitt’s lymphoma lines) relative to blood mononuclear cells [15]. In the present study, we show that EER-1 has a similar selective toxicity towards canine lymphoma cells relative to normal mononuclear cells. This finding suggest that VCP-targeted therapy will be as relevant to canine lymphoma as it will to the human disease, and further highlights the value of spontaneous canine lymphoma as a model for translational studies.

To investigate the mechanism of EER-1 action in CLBL-1 cells, we began by assessing its effects on ER stress. Wang et al. demonstrated that treatment of the mantle cell lymphoma line JEKO-1 with 10 μM EER-1 resulted in a dramatic increase in the expression of ER stress markers including *DDIT3* within 10 h [15]. These authors further showed that the ER stress-responsive transcription factors ATF3 and ATF4 participate in the transcriptional activation of the pro-apoptotic gene *NOXA*, suggesting that the induction of ER stress by EER-1 represents a major pathway through which it exerts its cytotoxic effect. Surprisingly, we were not able to find any evidence of increased ER stress (or alteration of the functioning of the aggresome-autophagy pathway) in CLBL-1 cells under the treatment conditions that we used, leading us to investigate additional VCP-regulated biological processes. Multiple studies in recent years have shown that VCP extracts ubiquitinated substrates from chromatin, and that interference with this activity results in protein-induced chromatin stress, the consequences of which include inadequate responses to DNA damage and genomic instability [12]. Here, we show that EER-1 treatment results in a rapid accumulation in DNA damage in CLBL-1 cells in a manner co-incident with the accumulation of Lys48 polyubiquitinated proteins in the cytoplasm and nucleus. We further show that this is accompanied by an induction of the TRP53/ATM-dependent signaling pathway and results in an increase in *Cdkn1a* expression, which in turn is a likely mediator of both the G1 cell cycle arrest and induction of apoptosis that were observed. Exactly how EER-1 treatment results in increased DNA damage remains to be determined. A recent study [42] has shown that the DNA damage recognition subunits DDB2 and XPC must be promptly removed from chromatin in a VCP-dependent manner during DNA excision repair. Reduced VCP activity results in prolonged retention of DDB2 and XPC, which in turn results in an attenuation of repair and causes chromosomal aberrations [42]. Further studies will be required to determine if a similar mechanism occurs in lymphoma cells in response to EER-1, if additional processes and mediators are involved in mediating EER-1 toxicity, as well as to verify that the “DNA damage” mechanism is also relevant to human lymphoid malignancies.

## Conclusions

This study validated VCP as a novel therapeutic target for canine lymphoma and identified a novel cellular mechanism of EER-1 action centered on the DNA repair response. Further studies are needed to determine the precise pathways that lead to DNA damage, TRP53 activation and to apoptosis. Although an unexpected mechanism of action was identified in this instance, the canine model nonetheless permits the evaluation of novel therapeutic targets in an immunocompetent host with a spontaneously occurring cancer, and will therefore, in our opinion, represent a valid and valuable system to study VCP as a therapeutic target in lymphoid malignancies.

## Additional file

Additional file 1: Figure S1. ER stress and autophagy positive controls. CLBL-1 cells were treated with A) Thapsigargin at 1 μM or B) Chloroquine at 50 μM for the indicated times. Vehicle = DMSO. Each lane represents one independant sample.

## Abbreviations

AA: Aggresome-autophagy pathway
CAD: chromatin-associated degradation
DMSO: Dimethylsulfoxide
EER-1: Eeyarestatin 1
ER: Endoplasmic reticulum
ERAD: Endoplasmic reticulum-associated degradation
FACS: Fluorescence activated cell sorting
MALT: Mucosal associated lymphoid tissue
MIQE: Minimum information for publication of quantitative real-time PCR experiments
NHL: Non-hodgkin’s lymphoma
PBMC: Peripheral blood mononuclear cell
PCR: Polymerase chain reaction
VC: Valosin containg-protein

## References

[1] Breen M, Modiano JF (2008). Evolutionarily conserved cytogenetic changes in hematological malignancies of dogs and humans–man and his best friend share more than companionship. Chromosome research : an international journal on the molecular, supramolecular and evolutionary aspects of chromosome biology.

[2] Marconato L, Gelain ME, Comazzi S (2013). The dog as a possible animal model for human non-Hodgkin lymphoma: a review. Hematol Oncol.

[3] Merlo DF, Rossi L, Pellegrino C, Ceppi M, Cardellino U, Capurro C (2008). Cancer incidence in pet dogs: findings of the Animal Tumor Registry of Genoa, Italy. J vet intern med / American College of Veterinary Internal Medicine.

[4] Valli VE, San Myint M, Barthel A, Bienzle D, Caswell J, Colbatzky F (2011). Classification of canine malignant lymphomas according to the World Health Organization criteria. Vet Pathol.

[5] Vail DM PM, Young KM, Withrow SJ, Vail DM, Page RL (2012). Canine lymphoma and leukemias. In : Withrow and MacEwan’s Small Animal Clinical Oncology.

[6] Johnston PB, Yuan R, Cavalli F, Witzig TE (2010). Targeted therapy in lymphoma. J Hematol Oncol.

[7] Chao MP (2013). Treatment challenges in the management of relapsed or refractory non-Hodgkin's lymphoma - novel and emerging therapies. Cancer manag res.

[8] Fessart D, Marza E, Taouji S, Delom F, Chevet E (2013). P97/CDC-48: proteostasis control in tumor cell biology. Cancer Lett.

[9] DeLaBarre BCJ, Kopito RR, Brunger AT (2006). Central pore residues mediate the p97/VCP activity required for ERAD. Mol Cell.

[10] Fernandez-Saiz V, Buchberger A (2010). Imbalances in p97 co-factor interactions in human proteinopathy. EMBO Rep.

[11] Boyault C, Gilquin B, Zhang Y, Rybin V, Garman E, Meyer-Klaucke W (2006). HDAC6-p97/VCP controlled polyubiquitin chain turnover. EMBO J.

[12] Vaz B, Halder S, Ramadan K (2013). Role of p97/VCP (Cdc48) in genome stability. Front genet.

[13] Ramadan K (2012). p97/VCP- and Lys48-linked polyubiquitination form a new signaling pathway in DNA damage response. Cell cycle (Georgetown, Tex).

[14] Wang Q, Li L, Ye Y (2008). Inhibition of p97-dependent protein degradation by Eeyarestatin I. J Biol Chem.

[15] Wang Q, Mora-Jensen H, Weniger MA, Perez-Galan P, Wolford C, Hai T (2009). ERAD inhibitors integrate ER stress with an epigenetic mechanism to activate BH3-only protein NOXA in cancer cells. Proc Natl Acad Sci U S A.

[16] Haines DS (2010). p97-containing complexes in proliferation control and cancer: emerging culprits or guilt by association?. Genes Cancer.

[17] Zhu WW, Kang L, Gao YP, Hei Y, Dong J, Liu Y (2013). Expression level of valosin containing protein is associated with prognosis of primary orbital MALT lymphoma. Asian Pac j cancer prev : APJCP.

[18] Yamamoto S, Tomita Y, Hoshida Y, Iizuka N, Kidogami S, Miyata H (2004). Expression level of valosin-containing protein (p97) is associated with prognosis of esophageal carcinoma. Clin cancer res: an official journal of the American Association for Cancer Research.

[19] Yamamoto S, Tomita Y, Hoshida Y, Iizuka N, Monden M, Yamamoto S (2004). Expression level of valosin-containing protein (p97) is correlated with progression and prognosis of non-small-cell lung carcinoma. Ann Surg Oncol.

[20] Yamamoto S, Tomita Y, Hoshida Y, Sakon M, Kameyama M, Imaoka S (2004). Expression of valosin-containing protein in colorectal carcinomas as a predictor for disease recurrence and prognosis. Clin cancer res: an official journal of the American Association for Cancer Research.

[21] Valle CW, Min T, Bodas M, Mazur S, Begum S, Tang D (2011). Critical role of VCP/p97 in the pathogenesis and progression of non-small cell lung carcinoma. PLoS One.

[22] Magnaghi P, D'Alessio R, Valsasina B, Avanzi N, Rizzi S, Asa D (2013). Covalent and allosteric inhibitors of the ATPase VCP/p97 induce cancer cell death. Nat Chem Biol.

[23] Chou TFLK, Frankowski KJ, Shoenen FJ, Deshaies RJ (2013). Structure–Activity Relationship Study Reveals ML240 and ML241 as Potent and Selective Inhibitors of p97 ATPase. Chem Med Chem.

[24] Seiser EL, Thomas R, Richards KL, Kelley MK, Moore P, Suter SE (2013). Reading between the lines: molecular characterization of five widely used canine lymphoid tumour cell lines. Vet Comp Oncol.

[25] Rutgen BC, Hammer SE, Gerner W, Christian M, de Arespacochaga AG, Willmann M (2010). Establishment and characterization of a novel canine B-cell line derived from a spontaneously occurring diffuse large cell lymphoma. Leuk Res.

[26] Strober W. Trypan blue exclusion test of cell viability. Curr Protoc Immunol. 2001; Appendix 3:Appendix 3B. 10.1002/0471142735.ima03bs21 18432654

[27] Milczarek GJ, Martinez J, Bowden GT (1997). p53 Phosphorylation: biochemical and functional consequences. Life Sci.

[28] Tsujimoto Y, Tomita Y, Hoshida Y, Kono T, Oka T, Yamamoto S (2004). Elevated expression of valosin-containing protein (p97) is associated with poor prognosis of prostate cancer. Clin cancer res : an official journal of the American Association for Cancer Research.

[29] Yamamoto S, Tomita Y, Hoshida Y, Nagano H, Dono K, Umeshita K (2004). Increased expression of valosin-containing protein (p97) is associated with lymph node metastasis and prognosis of pancreatic ductal adenocarcinoma. Ann Surg Oncol.

[30] Yamamoto S, Tomita Y, Hoshida Y, Takiguchi S, Fujiwara Y, Yasuda T (2003). Expression level of valosin-containing protein is strongly associated with progression and prognosis of gastric carcinoma. J clin oncol : official journal of the American Society of Clinical Oncology.

[31] Yamamoto S, Tomita Y, Hoshida Y, Toyosawa S, Inohara H, Kishino M (2004). Expression level of valosin-containing protein (VCP) as a prognostic marker for gingival squamous cell carcinoma. Ann oncol : official journal of the European Society for Medical Oncology / ESMO.

[32] Yamamoto S, Tomita Y, Nakamori S, Hoshida Y, Iizuka N, Okami J (2004). Valosin-containing protein (p97) and Ki-67 expression is a useful marker in detecting malignant behavior of pancreatic endocrine neoplasms. Oncology.

[33] Yamamoto S, Tomita Y, Nakamori S, Hoshida Y, Nagano H, Dono K (2003). Elevated expression of valosin-containing protein (p97) in hepatocellular carcinoma is correlated with increased incidence of tumor recurrence. J clin oncol : official journal of the Am Soc Clin Oncol.

[34] Boelens J, Lust S, Offner F, Bracke ME, Vanhoecke BW (2007). Review. The endoplasmic reticulum: a target for new anticancer drugs. In vivo (Athens, Greece).

[35] Perez-Galan P, Mora-Jensen H, Weniger MA, Shaffer AL, Rizzatti EG, Chapman CM (2011). Bortezomib resistance in mantle cell lymphoma is associated with plasmacytic differentiation. Blood.

[36] Liu Y, Ye Y (2011). Proteostasis regulation at the endoplasmic reticulum: a new perturbation site for targeted cancer therapy. Cell Res.

[37] Hardie DL, Johnson GD, Khan M, MacLennan IC (1993). Quantitative analysis of molecules which distinguish functional compartments within germinal centers. Eur J Immunol.

[38] Auner HW, Moody AM, Ward TH, Kraus M, Milan E, May P (2013). Combined inhibition of p97 and the proteasome causes lethal disruption of the secretory apparatus in multiple myeloma cells. PLoS One.

[39] Bursavich MG, Parker DP, Willardsen JA, Gao ZH, Davis T, Ostanin K (2010). 2-Anilino-4-aryl-1,3-thiazole inhibitors of valosin-containing protein (VCP or p97). Bioorg Med Chem Lett.

[40] Chou TF, Brown SJ, Minond D, Nordin BE, Li K, Jones AC (2011). Reversible inhibitor of p97, DBeQ, impairs both ubiquitin-dependent and autophagic protein clearance pathways. Proc Natl Acad Sci U S A.

[41] Le Moigne RWS, Soriano S, Valle E, Anderson DJ, Djakovic S, Menon MK (2014). CB-5083 is a novel first in class p97 inhibitor that disrupts cellular protein homeostasis and demonstrates anti-tumor activity in solid and hematological models. In: 105th American association for cancer research annual meeting.

[42] Puumalainen MR, Lessel D, Ruthemann P, Kaczmarek N, Bachmann K, Ramadan K (2014). Chromatin retention of DNA damage sensors DDB2 and XPC through loss of p97 segregase causes genotoxicity. Nat Commun.

