# Supplementary figures and images for: Pharmacological targeting of valosin containing protein (VCP) induces DNA damage and selectively kills canine lymphoma cells
Source: BMC Cancer. 2015 Jun 24;15:479. doi: 10.1186/s12885-015-1489-1 (PMC4479320; doi:10.1186/s12885-015-1489-1)

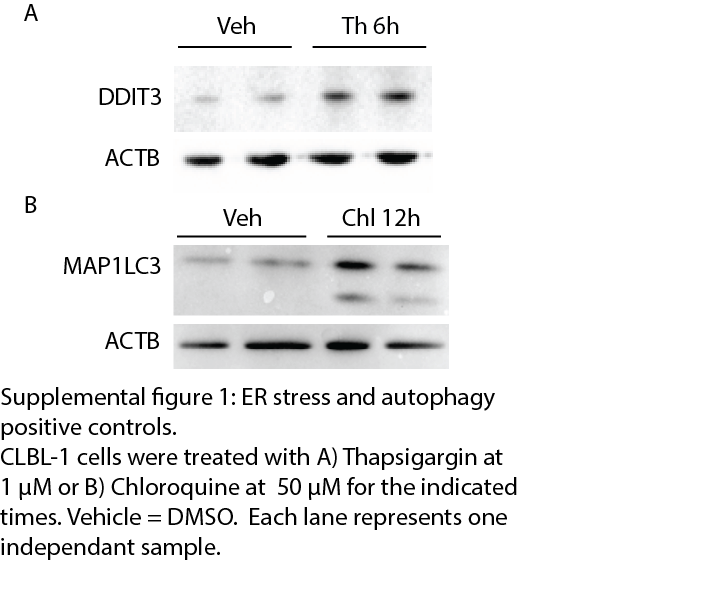

Supplement: Additional file 1: Figure S1. — ER stress and autophagy positive controls. CLBL-1 cells were treated with A) Thapsigargin at 1 μM or B) Chloroquine at 50 μM for the indicated times. Vehicle = DMSO. Each lane represents one independant sample. [file 12885_2015_1489_MOESM1_ESM.png]
